# Forecasting medical waste in Istanbul using a novel nonlinear grey Bernoulli model optimized by firefly algorithm

**DOI:** 10.1177/0734242X241271065

**Published:** 2024-09-09

**Authors:** Aziz Kemal Konyalıoğlu, Tuncay Ozcan, Ilke Bereketli

**Affiliations:** 1 Hunter Centre for Entrepreneurship, Strathclyde Business School, University of Strathclyde, Glasgow, UK; 2 Management Engineering Department, Faculty of Management, Istanbul Technical University, Istanbul, Turkey; 3 Industrial Engineering Department, Faculty of Engineering and Technology, Galatasaray University, Istanbul, Turkey

**Keywords:** Medical waste, grey forecasting, nonlinear grey Bernoulli model, firefly algorithm, parameter optimization

## Abstract

Waste management has gained global importance, aligning with the escalating impact of the COVID-19 pandemic and the associated concerns regarding medical waste, which poses threats to public health and environmental sustainability. In Istanbul, medical waste is considered a significant concern due to the rising volume of this waste, along with challenges in collection, incineration and storage. At this juncture, precise estimation of the waste volume is crucial for resource planning and allocation. This study, thus, aims to estimate the volume of medical waste in Istanbul using the nonlinear grey Bernoulli model (NGBM(1,1)) and the firefly algorithm (FA). In other words, this study introduces a novel hybrid model, termed as FA-NGBM(1,1), for predicting waste amount in Istanbul. Within this model, prediction accuracy is enhanced through a rolling mechanism and parameter optimization. The effectiveness of this model is compared with the classical GM(1,1) model, the GM(1,1) model optimized with the FA (FA-GM(1,1)), the fractional grey model optimized with the FA (FA-FGM(1,1)) and linear regression. Numerical results indicate that the proposed FA-NGBM(1,1) hybrid model yields lower prediction error with a mean absolute percentage error value 3.47% and 2.57%, respectively, for both testing and validation data compared to other prediction algorithms. The uniqueness of this study is rooted in the process of initially optimizing the parameters for the NGBM(1,1) algorithm using the FA for medical waste estimation in Istanbul. This study also forecasts the amount of medical waste in Istanbul for the next 3 years, indicating a dramatic increase. This suggests that new policies should be promptly considered by decision-makers and practitioners.

## Introduction

With the increasing global population and expanding industrial activities, awareness has grown regarding the consumption and production capabilities that generate various types of waste (Adamović et al., 2018; Çelik et al., 2023). Solid waste, resulting from both industrial and domestic activities, is a significant by-product of these processes (Ayeleru et al., 2021; Frosch, 1996). Medical waste, considered under solid waste, is particularly hazardous due to its toxic properties and is considered the second most dangerous type of waste after radioactive waste (Çetinkaya et al., 2020). This type of waste often contains harmful chemicals and serves as a potential medium for disease transmission (Dehghanifard and Dehghani, 2018; Komilis et al., 2012). Therefore, effective management of medical waste is crucial to prevent environmental pollution and mitigate health risks, including increased disease rates and other adverse health consequences (Gulec et al., 2001).

Inadequate management of medical waste can lead to severe environmental and health issues, including frequent floods, pest infestations and unpleasant odours (Turan et al., 2009). The scholars in medical waste management agree that medical waste, including pharmaceutical, hospital and infectious waste, poses significant health risks and contains harmful pollutants (Sabour et al., 2007). This issue is particularly pronounced in developing countries, such as Turkey, where ineffective medical waste management can result in financial losses, environmental degradation and serious health problems due to the inadequacies of municipal and governmental systems in managing this waste (Birpınar et al., 2009; Turan et al., 2009). Consequently, medical waste management remains a critical challenge in developing countries (Aghapour et al., 2013).

The COVID-19 pandemic has further highlighted the importance of managing medical waste, as the volume of healthcare waste (Hantoko et al., 2021), including masks, gloves and other medical materials, has surged in Turkey (Eren and Tuzkaya, 2021). In Turkey, alongside the overarching Regulation on Waste Management, specific legislation, known as the ‘Regulation on Medical Waste Control’, was initially enacted in 1993 and updated in 2005 and 2017 (Çakmak Barsbay, 2021; Coban et al., 2023). This regulation classifies infectious, pathological and sharps waste as ‘medical waste’ in Turkey (Coban et al., 2023; Eker et al., 2010; RG, 2017). The Turkish Ministry of Environment, Urbanization and Climate Change (MoEU) has issued various guidelines to enhance the implementation of this regulation, including those on medical waste sterilization, disposal and management during crises such as the COVID-19 pandemic (MoEU, 2017, 2020).

Despite these efforts, deficiencies in medical waste management practices persist in Istanbul, especially during crises (Balci et al., 2022; Polat, 2022). To address these issues, Sangkham (2020) proposed essential precautions for hospitals, practitioners and government entities, including the use of categorized containers, continuous operation and efficient collection and transportation procedures. Furthermore, scholars highlighted in this context that accurate forecasting of medical waste is vital for optimizing storage space, container design and vehicle routes for collection and distribution, thereby improving management efficiency during crises (Cao et al., 2023; Chen et al., 2021; Singh et al., 2022; Zhao et al., 2022).

Given that all risks inherently involve a degree of uncertainty, it is crucial to forecast factors and risks associated with medical waste (Eren and Tuzkaya, 2021). Despite its significant impact on daily life, the forecasting of medical waste generation has been insufficiently explored and modelled (Karpušenkaitė et al., 2018) especially in Turkey (Eren and Tuzkaya, 2021). This research thus seeks to address this gap by developing a hybrid model specifically designed to predict the amount of medical waste in Istanbul, utilizing grey forecasting models combined with metaheuristic algorithms, namely NGBM(1,1) and the firefly algorithm (FA).

The innovative aspect of this study is its foundational approach in optimizing the parameters of the NGBM(1,1) algorithm through the application of the FA. This optimization also aims to accurately estimate the quantity of medical waste in Turkey, with a particular focus on Istanbul. The optimized algorithm is subsequently employed to evaluate the amount of medical waste in the Istanbul region. The selection of NGBM(1,1), optimized by the FA, is based on its demonstrated high accuracy in previous forecasting studies (Khodabaccus and Saib, 2023; Ma et al., 2017, 2020).

Capitalizing on the distinct advantages of the FA and other metaheuristic algorithms, this study employs FA to optimize key parameters within NGBM(1,1) for the purpose of forecasting medical waste in Istanbul. Compared to alternative optimization techniques, the FA offers superior computational efficiency, requiring minimal prior knowledge, while providing enhanced robustness, self-adaptation and search capabilities. These characteristics are particularly beneficial for medical waste forecasting, as assessed by mean absolute percentage error (MAPE) metric.

The remainder of this study is organized as follows: the next section provides a detailed review of the literature on medical waste management and grey forecasting applications. The third section explains the nonlinear grey Bernoulli model (NGBM) and the FA. The fourth section presents the developed hybrid model for predicting the amount of medical waste, along with the dataset and application steps. The final section discusses the findings and conclusions.

## Literature review

### An overview of medical waste management

Numerous studies within the field of medical waste management exist in the literature. Aung et al. (2019) focused on evaluating medical waste management in accordance with World Health Organization guidelines. Their model, developed for this purpose, was implemented across eight diverse hospitals in Myanmar, encompassing both public and private institutions. Data collection involved surveys, interviews and observations, with multi-criteria decision-making methods employed to determine relevant waste management criteria. Stringent control measures are imperative for hazardous waste, encompassing adherence to regulations, laws, as well as leveraging experiential insights and technological advancements (Aung et al., 2019).

Zamparas et al. (2019) delved into the management of infectious waste in Greece. Their study involved a comparative analysis of related regulations across European Union countries, utilizing a multi-criteria model. The Analytic Hierarchy Process was applied from an environmental standpoint, with official data serving as input, and environmental management criteria yielding the most favourable outcomes (Zamparas et al., 2019).

Examining the biomedical waste generated during the COVID-19 pandemic, Ilyas et al. (2020) identified key sources such as facemasks, testing kits, personal protective equipment and gloves. The effective management of these waste streams is critical to curb the rapid spread of diseases. The study evaluated various applications under policy frameworks to recommend appropriate methods for the proper disposal of hazardous medical waste (Ilyas et al., 2020).

Sangkham (2020) conducted a medical waste study in the Asian region during the pandemic, underscoring the importance of adhering to guidelines and standards for hazardous waste disposal to mitigate environmental and social impacts. Tirkolaee et al. (2021) focused on infectious waste management during the COVID-19 pandemic, utilizing a mixed-integer linear programming model. This model addressed the disruption in regular waste management caused by the pandemic, aiming to minimize infection risks, travel times and deviations from time windows. The study applied this model in Iran, presenting diverse scenario results for managerial recommendations (Tirkolaee et al., 2021).

Singh et al. (2022) conducted a meta-analysis examining waste management in the context of the COVID-19 pandemic across different countries. Their investigation sought to understand the current state of medical waste management considering diverse socio-economic and environmental parameters. In another contribution to medical waste, Janik-Karpinska et al. (2023) explored various medical waste types to emphasize the significance of waste management for human health and environmental safety. Their article presented information on different management principles to offer a comprehensive overview of healthcare waste on a global scale, considering its potential harm to the population through various pathways (Janik-Karpinska et al., 2023).

### Forecasting applications by nonlinear grey Bernoulli modelling

Currently, NGBM finds applications in various forecasting scenarios. A comprehensive review of articles utilizing the NGBM(1,1) was conducted to identify diverse forecasting areas and analyses. Table 1 offers a summary of the pertinent articles that employ the NGBM approach for forecasting.

**Table 1.** Summary of NGBM applications in the literature.

Reference	Data source	Performance measures
Chen (2008)	Official data	RPE
Hsu (2010)	Official data	MAPE and RMSE
Wu et al. (2019)	Real-world data sets	MAPE
Şahin and Şahin (2020)	Official data	RMSE, MAPE, *R*^2^
Wang (2013)	Official data	RPE
Duman et al. (2019)	Official data	*R* ^2^
Xiao and Wang (2022)	Official data	MAE, MAPE, RMSE
Liu et al. (2022)	Official data	MAPE, APE
Xie et al. (2021)	Official data	MAPE, RMSE
Şahin (2021)	Official data	MAPE
Wu et al. (2021b)	Official data	MAPE
Jiang et al. (2021)	Official data	*R*^2^ and MAPE
Yang and Xie (2021)	Official data	MAPE, RMSE
Duan et al. (2021)	Official data	APE
Zheng et al. (2021)	Official data	MAE, RMSE, MAPE
Wang and Jv (2021)	Official data	MAE, RMSE, MAPE
Wang et al. (2011)	Official data	*R*^2^, MAPE
Wu et al. (2023)	Official data	MAPE, RMSE, NRMSE

MAE: mean absolute error; MAPE: mean absolute percentage error; NGBM: nonlinear grey Bernoulli model; RMSE: root mean square error; *R*^2^: coefficient of determination; RPE: relative performance metric; APE: absolute percentage error; NRMSE: normalized root mean square error.

The table compiles various studies retrieved from the literature, sourced through the Google Scholar and Web of Science databases. Within this literature, Chen (2008) utilized official unemployment rate data from 10 countries to assist governments in policy development. Hsu (2010) integrated GM(1,1) and the Grey Verhulst model to compare NGBM(1,1) results, forecasting the performance of the circuit industry in Taiwan. This study demonstrated that NGBM(1,1) is more accurate than GM(1,1) and the Grey Verhulst model. Wang (2013) employed NGBM(1,1) modelling in the finance field to estimate the main economic indices of high-tech enterprises in China. The conclusion was that NGBM(1,1) remains accurate even with a small sample size. Wu et al. (2019) introduced a novel model, FA-NGBM(1,1), incorporating fractional order accumulation to forecast various energy consumptions, including hydroelectricity, wind and solar.

In the healthcare domain, Şahin and Şahin (2020) highlighted a different application of NGBM(1,1) to forecast total COVID-19 cases in Italy and the USA. They also compared the results with GM(1,1) and FA-NGBM(1,1). Xie et al. (2021) estimated fuel combustion-related CO_2_ emissions using an integrated method, whereas Liu et al. (2022) optimized parameters using five different algorithms to apply NGBM(1,1) for estimating natural gas production in China.

In the energy consumption field, Şahin (2021) concluded that the NGBM yields accurate results with low errors when applied to renewable energy consumption in Spain, Germany, Turkey, Italy and the UK. Similarly, NGBM(1,1) was employed to forecast solar energy production in China by Wu et al. (2021b) and Jiang et al. (2021) attempted to estimate hydropower energy generation using NGBM(1,1) with a PSO algorithm-based parameter optimization. Yang and Xie (2021) presented a study to estimate coal consumption in China, emphasizing NGBM(1,1)’s high accuracy. Duan et al. (2021) integrated NGBM(1,1) with particle swarm optimization to predict global renewable energy consumption. Zheng et al. (2021) forecasted hydroelectricity consumption in the energy industry using an unbiased NGBM(1,1).

In the waste management field, NGBM(1,1) modelling has proven effective in forecasting waste. For instance, Wang et al. (2011) utilized NGBM(1,1) to estimate discharged wastewater in China. Duman et al. (2019) integrated NGBM(1,1) and particle swarm optimization to forecast electronic waste in Washington, comparing the results with multivariate grey models. Wang and Jv (2021) estimated solid waste and industry components by using NGBM(1,1) which yields high accuracy. Xiao and Wang (2022) used an improved NGBM(1,1) with particle swarm optimization of parameters to predict WEEE amount in China, concluding that the WEEE recycling industry’s development trend aligns accurately with the results and actual values. As one of the latest studies in 2023, Wu et al. (2023) applied NGBM(1,1) combined with an MPA-based optimization to predict petroleum consumption in China.

It is also noteworthy that several authors have articulated the advantages of grey forecasting models over alternative methods in the literature (Tseng et al., 2001; Wang and Jiang, 2019; Zeng et al., 2020). The advantages inherent in the grey forecasting model are robust compared to other machine learning and statistical methods including ARIMA, SARIMA, ANN, time series methods and SVM (Wang and Jiang, 2019; Wang et al., 2018; Wu et al., 2021a). Firstly, its adaptability shines in situations where data are scant; remarkable results have been attained with as few as four observations to predict outcomes in unknown systems (Xie and Wang, 2017). Secondly, its utilization of a first-order differential equation for system characterization is noteworthy. This feature enables the model to thrive even when confronted with sparse data, demonstrating that a minimal amount of discrete data points is ample for system characterization. As a consequence, the grey forecasting model emerges as a formidable tool, particularly adept for forecasting in competitive environments where decision-makers must rely on limited historical data (Chen and Wang, 2012; Es, 2021).

Despite numerous studies employing SVM, regression-based methods, ARIMA and basic grey models to estimate medical waste in Turkey across various years (Altin et al., 2023; Çetinkaya et al., 2020; Ceylan et al., 2020; Erdebilli and Devrim-İçtenbaş, 2022; Hanedar et al., 2022; Korkut, 2018), the utilization of NGBM, particularly optimized by metaheuristics, remains absent in the literature for estimating medical waste amounts in Turkey’s largest city. Furthermore, there is a lack of comparison with traditional statistical and basic grey models, indicating a gap that necessitates a more comprehensive approach. To date, no studies have investigated the quantity of medical waste generated in Istanbul during the COVID-19 period and the subsequent impact of the pandemic on medical waste volumes using the NGBM(1,1) optimized by metaheuristics in comparison to classical grey models. Moreover, to the best of our knowledge, the existing literature lacks any study forecasting medical waste in Istanbul within the time span of 2004–2023. This underscores the novelty and robustness of our method, providing a new contribution to the waste literature. In this regard, we contend that existing methods have not been optimized to forecast medical waste in Istanbul through the utilization of metaheuristics, such as the FA, the effectiveness and accuracy of which have been demonstrated in the literature (Khodabaccus and Saib, 2023; Ma et al., 2017, 2020). Hence, our novelty and originality possess dual dimensions: firstly, our methodology involving the NGBM(1,1) optimized by the FA to forecast medical waste generation in Istanbul; and secondly, our exploration of a new policy timeframe, contributing to the discourse in a novel manner.

## Methodology

As discussed in the introduction and literature review section, accurate forecasting of medical waste is essential for assessing the reliability of precautionary measures to mitigate associated risks (Ali and Ahmad, 2019; Awad et al., 2004). However, there is a notable gap in the existing research, as medical waste generation forecasting has not been widely analysed or modelled, despite its significant impact on daily life (Karpušenkaitė et al., 2018). To address this gap, our study introduces a novel hybrid model that predicts the amount of medical waste using grey forecasting models combined with metaheuristic algorithms, specifically NGBM(1,1) and the FA, in Istanbul that we present in this section.

The innovation of this research lies in our foundational approach to optimizing the parameters of the NGBM(1,1) algorithm using the FA. This optimization is specifically tailored to estimate the quantity of medical waste in Turkey, particularly in Istanbul. The optimized algorithm is then employed to assess the volume of medical waste in the Istanbul region. The rationale for using the NGBM(1,1) model optimized by the FA is its proven high accuracy in forecasting, as documented in previous studies (Khodabaccus and Saib, 2023; Ma et al., 2017, 2020).

In contrast to other optimization algorithms, the FA demonstrates superior computational efficiency, requiring minimal prior information. It also offers enhanced robustness, self-adaptation and search capabilities, which are particularly beneficial for forecasting medical waste, as evaluated by the MAPE metric. By leveraging the unique advantages of the FA and metaheuristic algorithms, our study significantly advances the methodology for medical waste forecasting, addressing a critical need in current research.

NGBM(1,1), FA and the proposed hybrid FA-NGBM(1,1) can be seen in this section respectively.

### Nonlinear grey Bernoulli model

The detailed steps and procedure of NGBM (1,1) model is explained as follows (Ding et al., 2021; Lu et al., 2016).

Step 1: Taking the original data by assuming that the collected observations with n entries are given as $X^{\left(0\right)}=\left\{x^{\left(0\right)}(1),x^{\left(0\right)}(2),\cdots ,x^{\left(0\right)}(n)\right\},n\geq 4$ , in which the datasets are all positives and equally spaced over time. Subsequently, the corresponding taken sequence is given by $Y^{\left(0\right)}=\left\{y^{\left(0\right)}(1),y^{\left(0\right)}(2),\cdots ,y^{\left(0\right)}(n)\right\},n\geq 4$ , obtained by using accumulated generating operation technique.
Step 2: Obtaining the intermediate series by the aid of applying one-time accumulated generating operation in order to obtain the intermediate series: $Y^{\left(1\right)}=\left\{y^{\left(1\right)}(1),y^{\left(1\right)}(2),\cdots ,y^{\left(1\right)}(n)\right\}$ , for which the k^th^ entry is defined as given in equation (1) (Ding et al., 2021).

(1) $$y^{\left(1\right)}\left(k\right)=\sum_{i=1}^{k}y^{\left(1\right)}\left(i\right),\;k=1,2,\cdots ,n.$$

Step 3: Constructing the grey differential equations and estimating the parameters to create the first-order grey differential equation of NGBM(1,1) and it can be defined as given in equation (2):

(2) $$y^{\left(0\right)}\left(k\right)+\beta_{1}z^{\left(1\right)}\left(k\right)=\beta_{2}{\left(z^{\left(1\right)}\left(k\right)\right)}^{\gamma },\gamma \neq 1,k=2,3,\cdots ,n.$$

Here, it should be noted that $y^{\left(0\right)}(k)$ is defined as the grey derivative, and $z^{\left(1\right)}(k)=0.5*\left(y^{\left(1\right)}(k)+y^{\left(1\right)}(k-1)\right)$ is identified as the background value of the grey derivative, and, $\beta_{2}$ are the generating parameters.

Furthermore, γ is to be referred to the power exponent serving as a nonlinear adjustable parameter endowing NGBM (1,1) model which provides an accurate flexibility and adaptability to estimate nonlinear sequences (Ding et al., 2021).

Now, let us assume that the power exponent is known and let us substitute the values of k into equation (2). Thus, one may see in equation (3).

(3) $$\begin{array}{l} y^{\left(0\right)}(2)+\beta_{1}z^{\left(1\right)}(2)=\beta_{2}{\left(z^{\left(1\right)}(2)\right)}^{\gamma }\\ y^{\left(0\right)}(3)+\beta_{1}z^{\left(1\right)}(3)=\beta_{2}{\left(z^{\left(1\right)}(3)\right)}^{\gamma }\\ \vdots \\ y^{\left(0\right)}(n)+\beta_{1}z^{\left(1\right)}(n)=\beta_{2}{\left(z^{\left(1\right)}(n)\right)}^{\gamma } \end{array}$$

The same equation can be given in the matrix form, Y=Zβ , as given in equation (4).

(4) $$Y=\left[\begin{array}{c} y^{\left(0\right)}(2)\\ y^{\left(0\right)}(3)\\ \vdots \\ y^{\left(0\right)}(n) \end{array}\right]Z=\left[\begin{array}{cc} -z^{\left(1\right)}(2) & {\left(z^{\left(1\right)}(2)\right)}^{\gamma }\\ -z^{\left(1\right)}(3) & {\left(z^{\left(1\right)}(3)\right)}^{\gamma }\\ \vdots & \vdots \\ -z^{\left(1\right)}(n) & {\left(z^{\left(1\right)}(n)\right)}^{\gamma } \end{array}\right]\beta =\left[\begin{array}{c} {\hat{\beta }}_{1}\\ {\hat{\beta }}_{2} \end{array}\right]$$

The matrix form given in equation (4) can be solved by using the least-squares estimation for $\beta_{1}$ and $\beta_{2}$ and $\beta ={\left[{\hat{\beta }}_{1},{\hat{\beta }}_{2}\right]}^{T}={\left(Z^{T}Z\right)}^{-1}B^{T}Y$ . Furthermore, one may see that ${\hat{\beta }}_{1}$ and ${\hat{\beta }}_{2}$ can also be calculated as given in equation (5)

(5) $$\begin{array}{c} {\hat{\beta }}_{1}=\frac{\sum_{k=2}^{n}{\left(z^{\left(1\right)}\left(k\right)\right)}^{\gamma +1}\sum_{k=2}^{n}y^{\left(0\right)}\left(k\right){\left(z^{\left(1\right)}\left(k\right)\right)}^{\gamma }-\sum_{k=2}^{n}{\left(z^{\left(1\right)}\left(k\right)\right)}^{2\gamma }\sum_{k=2}^{n}y^{\left(0\right)}\left(k\right)z^{\left(1\right)}\left(k\right)}{\sum_{k=2}^{n}{\left(z^{\left(1\right)}\left(k\right)\right)}^{2\gamma }\sum_{k=2}^{n}{\left(z^{\left(1\right)}\left(k\right)\right)}^{2}-{\left(\sum_{k=2}^{n}{\left(z^{\left(1\right)}\left(k\right)\right)}^{\gamma +1}\right)}^{2}}\\ {\hat{\beta }}_{2}=\frac{\sum_{k=2}^{n}{\left(z^{\left(1\right)}\left(k\right)\right)}^{2}\sum_{k=2}^{n}y^{\left(0\right)}\left(k\right){\left(z^{\left(1\right)}\left(k\right)\right)}^{\gamma }-\sum_{k=2}^{n}{\left(z^{\left(1\right)}\left(k\right)\right)}^{\gamma +1}\sum_{k=2}^{n}y^{\left(0\right)}\left(k\right)z^{\left(1\right)}\left(k\right)}{\sum_{k=2}^{n}{\left(z^{\left(1\right)}\left(k\right)\right)}^{2\gamma }\sum_{k=2}^{n}{\left(z^{\left(1\right)}\left(k\right)\right)}^{2}-{\left(\sum_{k=2}^{n}{\left(z^{\left(1\right)}\left(k\right)\right)}^{\gamma +1}\right)}^{2}} \end{array}$$

Step 4: Whitening differential equation can be built and solved based on the grey theory, the whitening differential function of NGBM (1,1) is illustrated as given in equation (6)

(6) $$\frac{dy^{\left(1\right)}\left(t\right)}{dt}+\beta_{1}y^{\left(1\right)}(t)=\beta_{2}y^{\left(1\right)}{\left(t\right)}^{\gamma },\gamma \neq 1$$

The parameters $\beta ={\left[{\hat{\beta }}_{1},{\hat{\beta }}_{2}\right]}^{T}$ can be substituted into equation (6), and the differential function in equation (6) can be solved to obtain the generalized solution in equation (7):

(7) $$y^{\left(1\right)}\left(t\right)={\left[Ce^{-\left(1-\gamma \right){\hat{\beta }}_{1}t}+\frac{{\hat{\beta }}_{2}}{{\hat{\beta }}_{1}}\right]}^{\frac{1}{1-\gamma }}$$

Here, it can be noted that C is a constant and by taking ${y^{\left(1\right)}(t)|}_{t=1}=y^{\left(1\right)}(1)$ as the initial condition and by substituting it into equation (7), we now obtain the equation (8):

(8). $$C=\left[{\left(y^{\left(0\right)}\left(1\right)\right)}^{1-\gamma }-\frac{{\hat{\beta }}_{2}}{{\hat{\beta }}_{1}}\right]e^{\left(1-\gamma \right){\hat{\beta }}_{1}}$$

Subsequently, the time response function of NGBM (1,1) model can be obtained as given in equation (9).

(9) $${\hat{y}}^{\left(1\right)}\left(k\right)={\left\{\left[{\left(y^{\left(0\right)}\left(1\right)\right)}^{1-\gamma }-\frac{{\hat{\beta }}_{2}}{{\hat{\beta }}_{1}}\right]e^{-\left(1-\gamma \right){\hat{\beta }}_{1}\left(k-1\right)}+\frac{{\hat{\beta }}_{2}}{{\hat{\beta }}_{1}}\right\}}^{\frac{1}{1-\gamma }}$$

### Firefly algorithm

In the firefly optimization algorithm, two critical considerations must be addressed: the variation in light intensity and the formulation of attractiveness (Gandomi et al., 2013). The attractiveness of a firefly is typically determined by its brightness, also referred to as light intensity, which is associated with the objective function (Gazi and Passino, 2004). Moreover, attractiveness is commonly assessed by the firefly’s visual perception, where light intensity decreases with distance from the source due to absorption in the medium (Gandomi et al., 2013). Let us define the light intensity, denoted as I(r) , according to the law of inverse square and absorption given in equation (10):

(10) $$I(r)=I_{0}\cdot e^{-\gamma \cdot r^{2}}$$

where $I_{0}$ and γ are subsequently explained as the original light intensity and light absorption coefficient (Gandomi et al., 2013). And the definition of attractiveness β(r) is given by the equation (11):

(11) $$\beta (r)=\beta_{0}\cdot e^{-\gamma \cdot r^{2}}$$

Here, it should be noted $\beta_{0}$ is expressed as the attractiveness when γisequalto0. To calculate the distance between any two fireflies i and j at $x_{i}$ and $x_{j}$ , one may use the Cartesian distance as given in equation (12):

(12) $$r_{ij}=\sqrt{\sum_{p=1}^{d}{\left(x_{i,p}-x_{j,p}\right)}^{2}},$$

where $x_{i,p}$ is donated as the p th component of the spatial coordinate $x_{i}$ of i th firefly (Gandomi et al., 2013).

Another issue is explained the concept of rand denoted as a *d*-dimensional uniform random vector given in [0 , $1{]}^{d}$ , and α defined as a parameter in the interval of [0,1] . Then, one may see that the new position $x_{i}$ of a firefly attracted by the brighter firefly j at time t+1 , can be followed as given in equation (13):

(13) $$x_{i,t+1}=x_{i,t}+\beta_{0}\cdot e^{-\gamma \cdot r_{ij}^{2}}\cdot \left(x_{j,t}-x_{i,t}\right)+\alpha \cdot (\mathrm{rand}-0.5)$$

Given that while the distance r is small value, the random term may avoid blockages in the local minima optimally. Contrary to this fact, if r is large, the firefly goes like a random walk.

FA is seen as being a powerful method for nonlinear design optimization problems (Koziel and Yang, 2011). The use of FA in this study can be stated as the fact that existing PSO and evolutionary algorithms do not show a performance as much as FA to search for the global optimum (Yang, 2010).

### Proposed approach: FA-NGBM(1,1) hybrid model

NGBM has two parameters such as production coefficient of the background value (α) and power index (γ). Correct adjustment of these parameters significantly affects the prediction quality. This problem can be formulated using the optimization model expressed by equations (14)–(16). In this model, production coefficient of the background value (α) and power index (γ) are decision variables, and the objective function is to minimize MAPE.

(14) $$\min\;Z=\frac{1}{n}\sum_{k=1}^{n}\left|\frac{{\hat{x}}_{0}(k)-x_{0}(k)}{x_{0}(k)}\right|\times 100\%$$

*s.t.*

(15) 0≤α≤1

(16) γ≠1

In equation (14), $x_{0}(k)$ indicates the actual value, ${\hat{x}}_{0}\left(k\right)$ indicates the predicted value and *n* is the number of test data.

In the proposed approach, the FA is used to solve the above parameter optimization problem. The pseudocode of the proposed hybrid FA-NGBM(1,1) model is presented in Table 2.

**Table 2.** The pseudo-code of the proposed FA-NGBM(1,1) hybrid method.


1: **Load** the dataset 2: **Divide** data into training, testing and validation datasets 3: **Initialize** firefly parameters (light absorption coefficient = 1, step factor = 0.01, β_0_ = 1 and β_min_ = 0.2) 4: **Define** α and γ parameters of NGBM(1,1) *randomly* 5: **Set** *t* = 1 6: Whilst (*t* < max number of iterations) 7: **Calculate** MAPE of NGBM(1,1) by using training data 8: **Set** fitness function = MAPE_i_ 9: Calculate MAPE_i_ 10: **if** (MAPE(*i* + 1) <MAPE*i*) **then** 11: **Update** α and γ 12: **end if** 13: *t* = *t* + 1 14: **end whilst** 15: Create a NGBM(1,1) model with finalized α and γ parameters 16: Calculate MAPE value of the test and validation data

FA: firefly algorithm; NGBM: nonlinear grey Bernoulli model; MAPE: mean absolute percentage error.

## Application

In this section, the dataset used, the application steps and performance analysis of the proposed approaches are presented with a real case study.

### Data set

**Figure 1.**
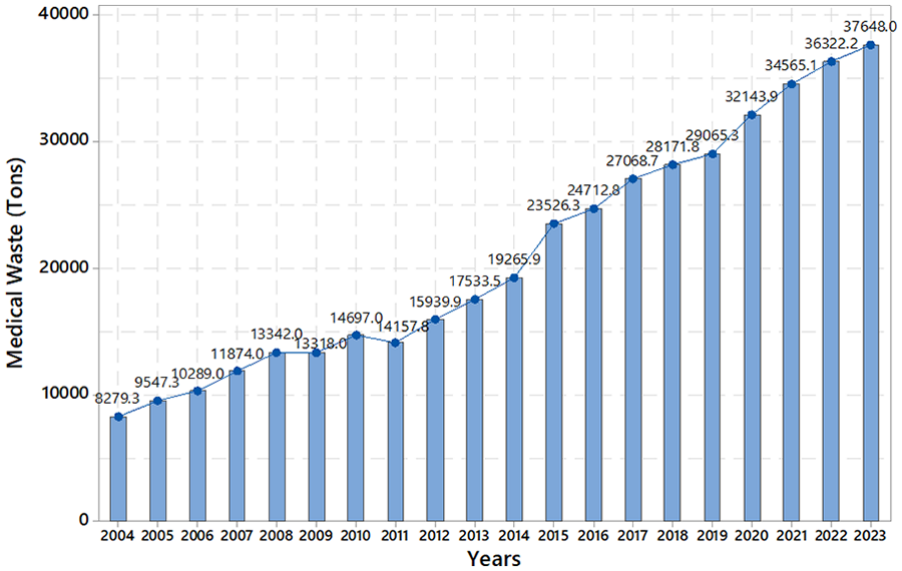
Medical waste amounts in Istanbul from 2004 to 2023.

The study focuses on Istanbul province, the largest city in Turkey. In Turkey, municipalities bear the responsibility for the collection, handling, transfer, incineration and disposal of medical waste, as per the regulations outlined in the Medical Waste Control Legislation governed by the government (MWCR, 2017). To conduct this study, 20 years’ worth of medical waste data was gathered from the Turkish Statistical Institute (https://www.tuik.gov.tr/Home/Index, accessed on 4 March 2024) and the Istanbul Metropolitan Municipality database (https://data.ibb.gov.tr/dataset?q=data, accessed on 4 March 2024). Both the Turkish Statistical Institute and Istanbul Municipality are key entities responsible for data collection. Figure 1 illustrates the medical waste generation in Istanbul from 2004 to 2023, encompassing the COVID-19 period. It is evident that the amount of medical waste in Istanbul experienced a significant increase during the COVID-19 period.

The dataset for Istanbul’s medical waste comprises annual data from 2004 to 2023 (*n* = 20). According to this dataset, the amount of medical waste in Istanbul exhibits substantial variation over time, increasing by approximately 4.5-fold from 2004 to 2023. Conversely, the number of hospital beds increased by approximately 1.5 times, and the number of doctors increased by approximately 1.7 times between 2004 and 2023 in Istanbul. Figure 1 shows the medical waste amounts in Istanbul from 2004 to 2023.

### Prediction of medical waste amount with proposed models

**Figure 2.**
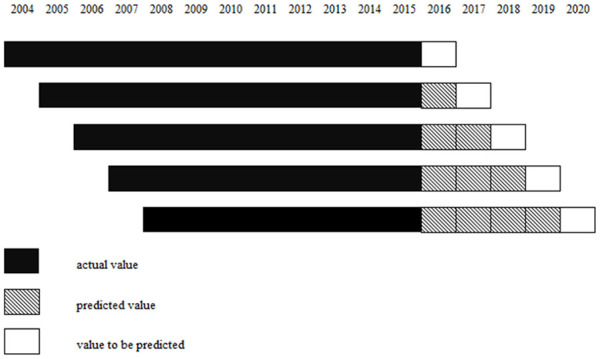
The diagram of the rolling mechanism in this study.

This study aims to predict the future medical waste (in tonnes) in Istanbul using a NGBM optimized with the FA (FA-NGBM(1,1)). Additionally, the performance of this proposed model is assessed by comparing it with GM(1,1), FA-GM(1,1), FA-FGM(1,1) and linear regression. The dataset is divided into 60% training (2004–2015), 25% testing (2016–2020) and 15% validation (2021–2023) (Baglaeva et al., 2020; Joseph, 2022). The rolling mechanism employed to generate these models is illustrated in Figure 2.

In the parameter optimization model, MAPE is used as the fitness function. The parameters of FA for the optimization model are as follows: the maximum iteration number is 1000, the population size is 50, the light absorption coefficient (γ) is 1, the step factor (α) is 0.01, β_0_ is 1 and the minimum attractiveness (β_min_) is 0.2. In the FA, MAPE converges very fast to a stationary point. The prediction models and parameter optimization problem are programmed on MATLAB 2022a. The prediction results and MAPE values obtained by the proposed models for testing data are given in Table 3.

**Table 3.** MAPE and predicted values of the proposed models for testing data.

Year	Actual value	Linear regression		GM(1,1)		FA-GM(1,1)		FA-FGM(1,1)		FA-NGBM(1,1)	
		Fitted value	Error (%)	Fitted value	Error (%)	Fitted value	Error (%)	Fitted value	Error (%)	Fitted value	Error (%)
2016	24712.81	21815.35	11.72	23395.68	5.33	23286	5.77	23290.57	5.76	23319.75	5.64
2017	27068.74	22969.38	15.14	25361.12	6.31	25185.57	6.96	25160.74	7.05	25230.63	6.79
2018	28171.81	24123.4	14.37	27468.77	2.50	27206.26	3.43	27134.44	3.68	27328.94	2.99
2019	29065.25	25277.43	13.03	29920.41	2.94	29539.15	1.63	29450.88	1.33	29625.44	1.93
2020	32143.85	26431.46	17.77	32791.42	2.01	32249.84	0.33	32196.32	0.16	32148.85	0.02
MAPE(%) 2016–2020			14.41		3.82		3.62		3.60		3.47

MAPE: mean absolute percentage error; FA: firefly algorithm; NGBM: nonlinear grey Bernoulli model.

In the proposed FA-NGBM(1,1) model, the minimum MAPE (3.47%), α = 0.88 and γ = −0.42 are obtained by using FA. Similarly, the minimum MAPE (3.62%) and α = 0.53 are found with the parameter optimization in the FA-GM(1,1) model. In addition, in the fractional grey model with FA (FA-FGM(1,1)), minimum MAPE (3.60%), α = 0.21 and *r* = 0.98 are calculated.

According to the results in Table 3, the proposed hybrid FF-NGBM(1,1) has the minimum MAPE value of 3.47%, whereas linear regression model has the highest MAPE value of 14.41%. The numerical results also indicate that parameter optimization and rolling strategy improves the forecasting accuracy of GM(1,1), FGM(1,1) and NGBM(1,1) models.

For validation of the proposed models, the dataset of the last 3 years is used. Prediction models are run with optimized parameter values using training data and rolling mechanism. The performance analysis of the prediction models for the validation data is presented in Table 4.

**Table 4.** MAPE and predicted values of the proposed models for validation data.

Year	Actual value	Linear regression		GM(1,1)		FA-GM(1,1)		FA-FGM(1,1)		FA-NGBM(1,1)	
		Fitted value	Error (%)	Fitted value	Error (%)	Fitted value	Error (%)	Fitted value	Error (%)	Fitted value	Error (%)
2021	34565.10	27585.49	20.19	35454.39	2.57	35252.98	1.99	35535.79	2.81	34396.90	0.49
2022	36322.21	28739.52	20.88	38426.32	5.79	38130.16	4.98	38559.71	6.16	36945.43	1.72
2023	37648.01	29893.55	20.60	41622.98	10.56	41207.30	9.45	41812.43	11.06	39725.36	5.52
MAPE(%) 2021–2023			20.56		6.31		5.47		6.68		2.57

MAPE: mean absolute percentage error; FA: firefly algorithm; NGBM: nonlinear grey Bernoulli model.

As can be seen from Table 4, the proposed FA-NGBM(1,1) model has a prediction error of 2.57% for validation data. The proposed model has better performance than other prediction models such as linear regression, GM(1,1), FA-GM(1,1) and FA-FGM(1,1).

**Figure 3.**
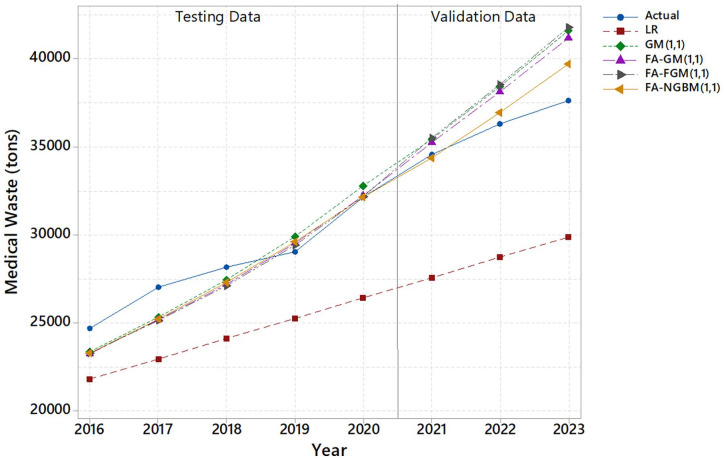
Performance analysis of the prediction models for the testing and validation data.

The performance analysis of the prediction models for the testing and validation data is presented visually in Figure 3.

Finally, the amount of waste is estimated for 2024, 2025 and 2026 using the FA-NGBM(1,1) model, which has the lowest prediction error in the validation and test data. Prediction results for future 3 years are presented in Table 5. These prediction results provide guidance for policymakers in terms of resource planning and management.

**Table 5.** Waste amount predictions for future years with the FA-NGBM(1,1) model.

Year	2024	2025	2026
Medical waste (tonnes)	41096.34	43825.85	46779.47

FA: firefly algorithm; NGBM: nonlinear grey Bernoulli model.

## Discussions, limitations and further research

### Discussions

The heightened demand for medical materials and associated services has led to a substantial increase in waste generation, particularly infectious medical waste (IMW) contaminated with viruses (Dharmaraj et al., 2021). This waste includes items such as masks, protective clothing, pharmaceutical packaging and domestic waste produced during prevention, control, treatment and recovery activities. The management of IMW is crucial due to its potential risks to human health. Several authors have emphasized that accurately forecasting future medical waste (MW) generation is essential for establishing an effective MW management system. Such forecasting informs decisions regarding the size of storage facilities, the number and type of collection equipment and future treatment and disposal capacity needs (Ceylan et al., 2020; Fadaei, 2023; Samastı et al., 2024; Torkashvand et al., 2022). Despite the recent increase in treatment facilities in Turkey, MW management issues persist and the rising population and socio-economic development in Turkey contribute to significant MW generation, with Istanbul’s population of approximately 16 million generating substantially more MW than other cities. Thus, research into suitable models to explain MW generation data is vital for successful MW management in metropolitan areas like Istanbul (Çelik et al., 2023; Görçün et al., 2023).

Accurately forecasting future MW generation is imperative for implementing an efficient MW management system. This includes determining storage facility capacity, specifying collection equipment and anticipating capacity requirements for treatment and disposal facilities. Karpušenkaitė et al. (2018) highlighted that the core concept of medical waste forecasting revolves around predicting future quantities of specific types of medical waste based on historical and current data. Analysing factors contributing to fluctuations in medical waste generation over time allows for better anticipation of future waste management sector needs (Golbaz et al., 2019). This proactive approach enables accurate calculations and targeted investments in projects such as waste incineration plants, recycling facilities and manufacturing from sorted materials. These investments can yield significant cost savings for taxpayers, promote environmental cleanliness and stimulate job creation in industries focused on waste assimilation methods (Karpušenkaitė et al., 2016).

Despite the establishment of treatment facilities in Turkey, challenges persist, especially in megacities like Istanbul. To address this, our study introduces a hybrid method, FA-NGBM(1,1), essential for effective medical waste management during crises such as the COVID-19 pandemic. This study’s novelty lies in utilizing the FA to optimize parameters in the NGBM(1,1) algorithm, a novel approach in the literature. In the parameter optimization model, the MAPE serves as the fitness function, with specific parameters for the FA. The proposed FA-NGBM(1,1) model achieves a minimum MAPE of 3.47% with optimized parameters (α = 0.88, γ = −0.42). Similarly, the FA-GM(1,1) model attains a minimum MAPE of 3.62% with parameters (α = 0.53) through optimization. Additionally, the FA-FGM(1,1) model achieves a minimum MAPE of 3.60% with parameters (α = 0.21 and *r* = 0.98) optimized. In addition, this study forecasts medical waste amount in Istanbul for 2024, 2025 and 2026 by using our hybrid algorithm to suggest practical implications for decision-makers and practitioners.

As seen in Table 5, the medical waste amount in Istanbul will dramatically increase, which will rise up to 46779.47 tonnes. Thus, the practical implications of our research extend to waste management policies in Istanbul, providing compelling evidence that the COVID-19 pandemic has also significantly increased medical waste quantities in the city. Our study highlights inefficiencies during crisis periods and suggests that medical waste disposal will continue to escalate as seen in Table 5, influenced by various factors (Özgüven and Okur, 2022). This necessitates new regulations concerning transportation routes, capacity management, disposal methods and facility enhancements by the municipality of Istanbul and the Turkish government. Understanding waste forecasting and management during emergencies can enhance effectiveness in Istanbul, not only during COVID-19 but also in future crises. Accurate prediction and management of medical waste are imperative to address emerging challenges effectively.

Furthermore, our study identifies critical insights regarding medical waste management in Istanbul, particularly due to the COVID-19 pandemic. Based on these findings, we propose targeted policy recommendations to enhance the efficiency, effectiveness and sustainability of medical waste management practices. Given the multifaceted challenges in Istanbul, we suggest for developing and implementing comprehensive local regulatory frameworks tailored to the city’s unique dynamics, including crisis periods such as anticipated earthquakes. Effective management of medical waste becomes even more critical in such events, requiring proactive measures to mitigate public health and environmental impacts (Ergün Konukcu, 2023; Orak et al., 2023). Regulations targeting crisis periods should form a cornerstone of a resilient medical waste management infrastructure, providing clear directives for segregation, collection, transportation, treatment and disposal across healthcare facilities and waste management entities.

As a supplementary strategy, we advocate for comprehensive capacity-building initiatives tailored to medical waste management needs during crises and pandemics (Kargar et al., 2020). These initiatives should include capacity increases in facilities, educational programmes and professional development opportunities for stakeholders. By investing in stakeholder preparedness, including municipalities, health workers and governmental entities, Istanbul can enhance resilience to emergencies, minimize disruptions to waste management operations and mitigate risks to public health and environmental integrity during crises.

### Limitations and further research

This study has several inherent limitations that need acknowledgement. Firstly, not all parameters relevant to estimating medical waste in Istanbul, such as waste generation and collection from private institutions, were included. This omission may result in overlooking a small amount of data, potentially impacting the model’s accuracy. Moreover, during the COVID-19 period, challenges in data management could introduce deviations or lead to missing inputs, influencing the outcomes of our hybrid model.

Secondly, while our model appears effective, we did not explore alternative error calculation techniques like mean absolute deviation, mean absolute error, mean absolute relative error, mean square error, nonlinear autoregressive, partial least squares, relative mean errors (RME), root mean square error (RMSE) or coefficient of determination (*R*^2^), which are commonly used in the literature. Future research endeavours could benefit from investigating the optimization of the NGBM(1,1) model using different metaheuristic algorithms, including ant colony optimization, artificial bee colony or grey wolf optimization. Additionally, enhancing the study by developing multivariate grey models (GM(1,*N*)) that incorporate variables such as the number of hospital beds, medical centres, bed occupancy, pharmacy branch count and daily outpatient numbers could provide valuable insights, considering other error calculations such as *R*^2^, RMSE or RME.

## Conclusions

Waste management has gained global significance amid the escalating impact of the COVID-19 pandemic, particularly in handling medical waste, which poses dual threats to public health and environmental sustainability. Furthermore, predicting future MW generation is imperative for designing an effective MW management system, informing decisions on storage facility sizing, collection equipment types and quantities, as well as estimating future treatment and disposal capacity needs. In this context, the escalation of population growth and socio-economic advancements in Turkey markedly amplifies the generation of MW, with Istanbul, boasting a population of around 16 million, emerging as a major contributor to MW production compared to other urban centres nationwide. Hence, the exploration of an appropriate model elucidating MW generation data assumes critical importance for the effective management of medical waste in metropolitan areas such as Istanbul. Moreover, Istanbul confronts a mounting challenge in effectively managing the burgeoning volume of medical waste, characterized by inherent complexities in collection, incineration and storage processes. This study, thus, introduces a novel hybrid model, FA-NGBM(1,1), for predicting waste amounts in Istanbul. The performance of the proposed hybrid model is compared with classical GM(1,1), FA-GM(1,1), FA-FGM(1,1) and linear regression. Numerical results reveal that the FA-NGBM(1,1) model, incorporating a rolling mechanism, provides satisfactory predictions for medical waste amounts in Istanbul. Grey forecasting models demonstrate robust predictive capabilities, particularly in scenarios with limited data. Furthermore, parameter optimization, combined with the rolling mechanism, significantly enhances the performance of grey forecasting models like GM(1,1), FGM(1,1) and NGBM(1,1). The FA proves highly effective in terms of solution quality and runtime for solving the parameter optimization problem.

For future research, the optimization of the NGBM(1,1) model using other metaheuristic algorithms, such as ant colony optimization, artificial bee colony or grey wolf optimization, can be explored. Additionally, developing multivariate grey models (GM(1,*N*)) that incorporate variables such as the number of hospital beds, medical centres, bed occupancy, pharmacy branch count and the number of daily outpatients could offer further insights.
